# Cultural Differences and Importance of Somatic Symptoms Could Provide Hidden Clues and Potential Management Options for Depressed Patients with Suicidal Ideation

**Published:** 2013-06

**Authors:** Thomas I. Lemon

**Dear Editor,**

Seifsafari Sh, Firoozabadi A, Ghanizadeh A, Salehi AR. A Symptom Profile Analysis of Depression in a Sample of Iranian Patients during 2011. **Iranian Journal of Medical Sciences**, North America, 38, mar. 2013. Available at: <http://ijms.sums.ac.ir/index.php/ijms/article/view/832/399>. Date accessed: 12 Mar. 2013.

Firstly, I thank Seifsafari, Firoozabadi, Ghanizadeh, and Salehi for their interesting and relevant study.^1^ In this they conclude that somatic symptoms have a central part in the presentation of depressed patients and they urge physicians to not overlook the importance of such symptoms when synthesising their diagnosis and management plan. I agree it is incredibly easy for physicians who are not involved routinely with depressed patients to overlook some of the obvious presenting symptoms of depression both somatic and non-somatic (loss of interest in enjoyable activities, sleep change, etc.), and would imagine this is more likely to be the case in cultures where depression is on the ‘taboo spectrum’. 

The authors had an impressive sample of 500, all of whom were interviewed within one centre. Interviewing in one centre has pros and cons. Firstly, its likely to reduce operator variance as fewer doctors would have been involved in the assessment of these patients, but cons of a single institution importantly include protocol difference, which may exist between separate institutions and may have an effect on the rate of correctly identified patients–that is to say, many junior doctors have strict protocols to work to, and their ability to elicit certain findings is to some extent dependent on these. With further respect to methodology, Seifsafari et al. aimed to use psychiatric interview as the variable for assimilations with various demographic factors. This seems to have worked well to have given relevant and generalizable results.

For my part, the most interesting finding of Seifsafari et al.^1^ is the lack of difference in suicidal ideation between males and females, which the authors have found. As the authors correctly describe, in the Western world major depressive episodes are believed to be more common in males. Other notable risk factors include drug and alcohol abuse, low intellectual quotient (IQ), and lower social standing.^2^ To draw on this further, a recent study carried out by ourselves describes the importance of support for depressed substance users in the United Kingdom, due to their increased risk of suicidal ideation.^3^ We describe how a broader range of medical staff needs to be trained to have further skills to deal with this patient demographic, ‘widening the net’ for depression detection so to speak. In our study, we did not examine any sex differentiation and in light of the Seifsafari *et al*.^1^ study, this is perhaps something we should have done.

Depressive symptoms are, of course, well known to involve an increased risk in myocardial infarction^3^ and many other illnesses, so it is logical to expect many depressive episodes to present with some somatic symptoms, and it falls on the physician to differentiate this from general medical conditions, and also somatisation disorder. Somatisation disorder is more prevalent in females (2% female compared to 0.2% male), and hence somatic symptoms in men should be further highlighted as potential underlying depression or general medical complaint.^4^ One of the theories surrounding somatisation disorder is that it occurs due to a heightened sensitivity to internal physical conditions. Reduced serotonin and increased cortisol found in depression will cause effects on body organs, and as such result in somatic symptoms.^5^ Therefore, it is possible for somatisation disorder to in fact be physiologically linked to depression and as such should form part of the diagnostic workup.

This difference in sex and suicidal ideation that Seidsafari et al*.*^1^ have highlighted warrants further investigation, namely because as the authors describe briefly, this may be due to cultural differences. Perhaps when comparing Iranian and Western cultural differences, we could determine whether Iranian culture is providing prevention of suicidal ideation to men, or increased suicidal ideation to women, compared to the Western world. The answer to this question could be of huge relevance to depression management. It would further enable treatment to focus on adjusting to a different style of living than the typical Selective Serotonin reuptake inhibitors that we prescribe to this patient set. 

In summary, the authors have highlighted the importance of symptoms in depressed patients, which are sometimes overlooked. It is very easy to put these somatic symptoms down to factitious disorders, and overlook depression, but the epidemiology of such factitious disorders suggests that using this approach results in overlooking patients potentially at risk. It has also been highlighted that it is important to further analyse suicidal ideation and sex and for further comparisons to be drawn.
